# Feminization Surgery of the Upper Face as the Crucial Factor in Gender Confirmation—Pearls and Pitfalls

**DOI:** 10.3390/medicina60010120

**Published:** 2024-01-09

**Authors:** Rafał Pokrowiecki, Barbora Šufliarsky, Maciej Jagielak

**Affiliations:** 1 Prive Esthetic and Facial Feminization Surgery Centre, 02-640 Warsaw, Poland; 2 Department of Oral and Maxillofacial Surgery, Faculty of Medicine, Comenius University in Bratislava and University Hospital, 81372 Bratislava, Slovakia; 3 Orthognathic, Private Practice, 05-090 Warsaw, Poland; recepcja@ortognatyka.pl

**Keywords:** facial feminization, gender affirmation surgery, frontal sinus setback, plastic surgery, craniofacial surgery

## Abstract

*Background*: Upper-face feminization is a frequently executed procedure in sexual reassignment surgery, owing to its ability to influence gender identity through adjustments to the hairline, forehead, and peri-orbital area. The procedure includes reducing the hairline, lifting the brows, shaving the orbital region, and applying specific techniques to reduce the frontal bone. This research aims to assess the outcomes, results, and potential complications associated with this surgery among transgender patients. *Material and Method*: Retrospective review of medical records of 20 patients who attended for facial feminization surgery of the upper face between June 2022 and June 2023, analyzing the previously performed procedures, complications and revision surgery outcomes, and first-time procedures. A literature review was performed for similar studies. *Results*: 20 patients were included in the study. Among the cohort treated elsewhere (*n* = 11), the primary complaint was insufficient browbone reduction and anterior frontal sinus table setback. They underwent poorly performed Type 1 reduction when full forehead reconstruction (Type 3/4) was indicated (*n* = 3), or no reduction was performed during hairline advancement (*n* = 4). Type 3 forehead reduction with orbital shaving and hairline advancement with simultaneous temporal browlift was most commonly performed in both revision and first-time surgical upper face feminization (*n* = 15) (75%). Type 1 osteoplasty was performed in four patients (10%), one Type 3 revision surgery was performed after insufficient Type 3 reduction, and one case of shock-induced alopecia was reported, treated with PRP/peptides and a FUE hair graft. *Conclusions*: The author’s preferred technique, ‘whole-in-one’ upper face feminization by modified bi-coronal incision with frontal trichophytic hyper-beveled incision, provides sufficient insight into the frontal bone and orbital region, the desired forehead osteoplasty and the most efficient insight into the temporal area, enabling safe dissection between fasciae, ligamentous adhesion removal, and periosteal attachment, providing full soft and hard tissue feminization. Nevertheless, feminization procedures should be meticulously planned, and all concerning issues should be addressed during the first surgery in order to prevent revisions, complications, and patient dissatisfaction.

## 1. Introduction

Gender dysphoria is defined as a state where gender identity is incongruent with the sex assigned at the patient’s birth. As facial characteristics are very important in gender recognition, feminization surgery is a complex and sequential set of procedures that enable to re-shape the facial features into something more feminine at both the bone- and soft tissue level [1,2]. As such, they are targeted at the distinctive anatomical differences seen between the anthropomorphic male and female skulls. Over 90% of trans-gender patients are in male-to-female transition (MTF). Among other surgeries that are necessary for gender confirmation surgery (e.g., breast augmentation, vaginoplasty, and waist-narrowing surgery), facial feminization surgery (FFS) is one of the most important phases throughout this process, as it is the face that defines the gender in the first place. Acquiring desired features matching the gender improves the self-esteem and social life of these patients, but it also decreases suicidal ideation, psychological distress, alcohol abuse, and smoking. Forehead and brow position are two of the most important factors in gender confirmation surgery, as the upper face represents 35–40% of the face [3,4]. Therefore, forehead feminization, along with rhinoplasty and jawline reshaping, is most commonly chosen by individuals undergoing gender affirmation surgery. Routinely, upper third feminization procedures involve hairline lowering and reshaping, different variants of browlift, reduction of the frontal bossing, and orbital aperture widening [5,6,7]. Protocols and surgery approaches may differ between the surgery centers providing gender-affirmation procedures. The fronto-glabellar region is the most challenging aspect of facial feminization in the upper third of the face. Douglas Ousterhout was the first to describe a detailed protocol for reshaping the forehead, taking into consideration the frontal bone and sinus anatomy. Type 1 forehead feminization involves the reduction of the frontal cortical bone. This procedure is suitable when the frontal sinus is underdeveloped and the cortical bone is thick enough to achieve both aesthetic goals and bone wall integrity. It typically allows for up to a 10 mm reduction in bone. Type 2 is performed when no setback is needed, but augmentation of the forehead crease is required to create a more feminine slope. Type 3 involves the reconstruction of the forehead with bone setback and stabilization using wires (following Ousterhout’s original recommendations) or titanium plates (as in current approaches). Type 4 forehead reconstruction is relatively rarely necessary and involves complete forehead augmentation. This is done when the frontal area is too narrow or the other three types are insufficient to achieve the desired aesthetic [1,3].

The aim of this study was to analyze treatment needs and surgery outcomes in a cohort of transgender patients who recently underwent upper-face feminization procedures. This is also the first multidisciplinary Central-East Europe FFS Team annual report on transfeminine facial surgery.

## 2. Materials and Methods

A retrospective review of the medical records of patients who attended for facial feminization surgery between June 2022 and June 2023. Inclusion criteria included patients undergoing facial gender-affirming surgery, including the fronto-orbital region, at least 6 months of actual hormone replacement therapy, and patients under the continuous care of a psychologist and sexologist. Exclusion criteria included incomplete files, patients undergoing facial surgeries not including the upper face, and adolescents. The study aimed at analyzing the previously performed procedures in the upper face performed elsewhere, complications, and reasons for revision surgery. This study was conducted in accordance with the principles of Helsinki. Written informed consent was obtained from the individuals whose pictures are presented within the manuscript depicted in this article.

## 3. Results

Among the 35 transfeminine patients who attended for facial feminization surgery between June 2022 and June 2023 20 were included in the study (Table 1). 11 individuals opted for revision surgery of the fronto-orbital region due to a lack of satisfaction after previous procedures. 15 patients were not included in this study as the FFS procedures planned did not involve the upper face. Common complaints reported by patients were: insufficient reduction/setback of the anterior table of the frontal sinus (*n* = 7), inadequate or lack of orbital rim widening (*n* = 8), inadequate or not modified/lowered hairline (*n* = 7) (Table 1).

**Figure 1:**
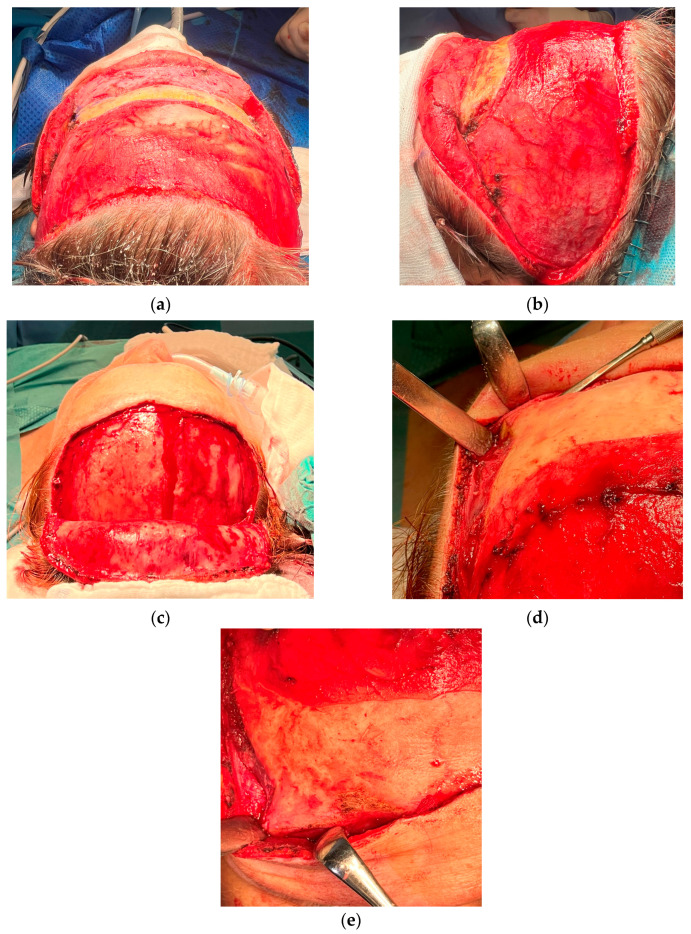
Examples of incision designs and flaps preparation commonly used in our protocol. (**a**,**b**) coronal pretrichial incision recommended in hairline lowering surgery and receding hairline reduction, as well as temporal browlift. This approach was performed in vast majority of the cases. (**c**–**e**) limited pretrichial when hairline lowering is advised but no intervention in temporal area is required. This approach provides comparable possibilities for insight into the peri-orbial area. Both approaches enable midface lift along with canth oplasty and cat eye surgery if desired. We usually do not use classic coronal incision (not shown here) as it does not provide sufficient feminization effects. We use this approach in cases with bone recontouring only and/or in cases without a need to address the hairline. However, coronal approach may also provide temporal and midface lifts if desired.

**Figure 2:**
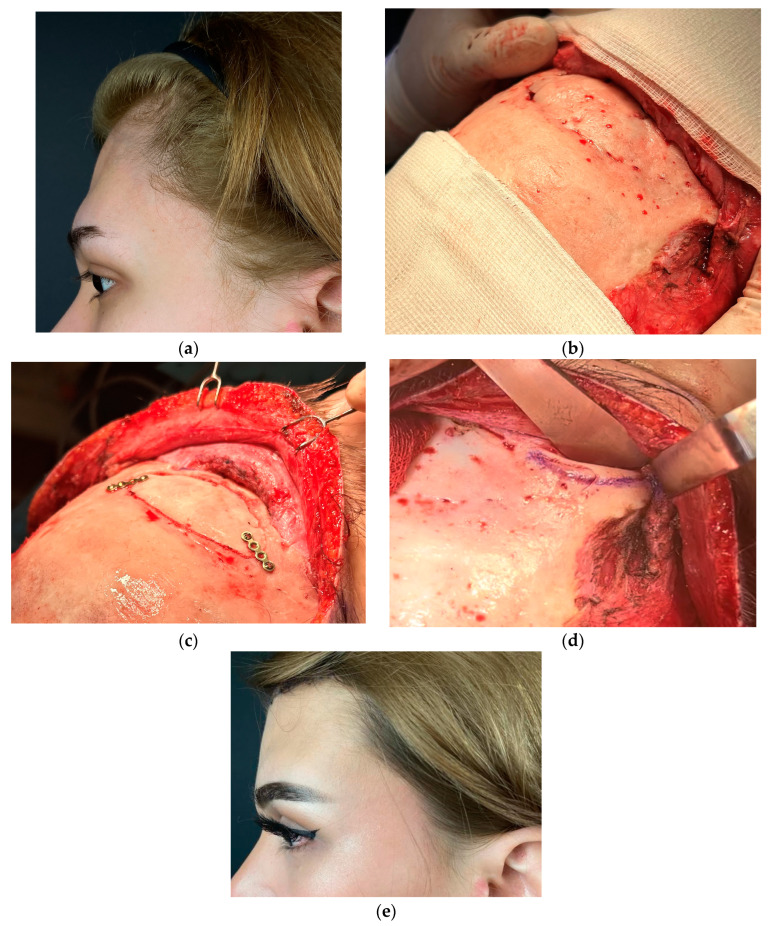
Photographs of the 19-year-old transgender female who attended revision surgery on the upper third. She complained of residual deformity, high hairline, and inadequate browlift. (**a**) lateral view of the forehead. (**b**) intraoperative view of the deformity and bone segment stabilized with Prolene 3.0 suture. (**c**) anterior wall repositioned and stabilized with 1.5 mm titanium plates (ChM, Lewickie, Poland). (**d**) Additional widening orbital rim ostectomy was performed. (**e**) lateral view 10 days post-op.

**Figure 3:**
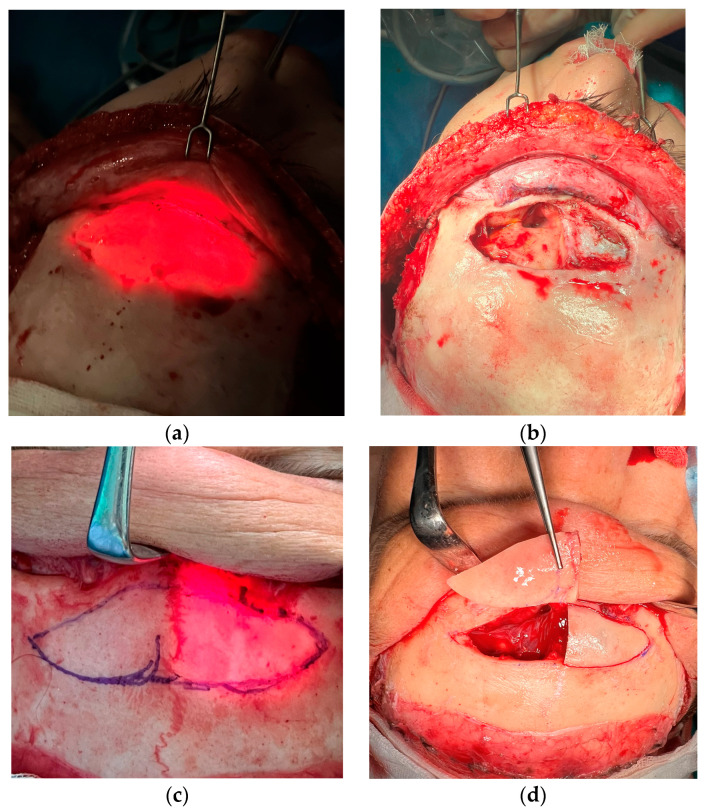
Intraoperative photograph of endoscopic-assisted transillumination technique of the frontal sinus that enables safe marking of the sinus border before resection and reposition. (**a**) large sinus without septum, where one block resection of the anterior wall was performed. (**b**) view of the frontal sinus after removal of the anterior table. (**c**) Transillumination of the bifid frontal sinus septum. (**d**) two-piece resection of the anterior wall was performed due to thick septum and risk of fracture.

**Figure 4:**
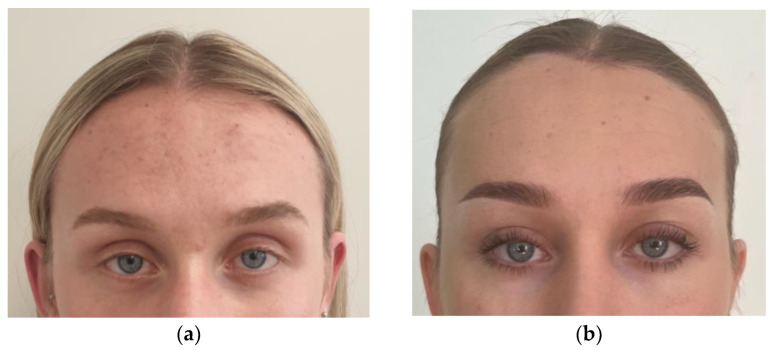

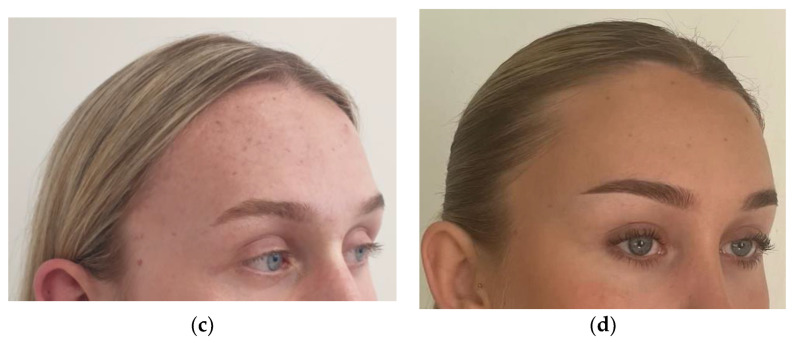
Photographs of 21-year-old transgender females after primary complex feminization of the upper third: Type 1 frontal bone reduction, extended orbital aperture widening, hairline lowering, temporal browlift. (**a**) before and (**b**) 6 months after the surgery. (**c**) before the surgery, and (**d**) 6 months pot-op.

A total of 9 patients attended our FFS Team for the first time for consultation, according to the detailed protocol and decision tree (Table 1, Table 2 and Table 3). A classical coronal pretrichial incision was performed in all the patients that required revision surgery. In 3 cases, a limited pretrichial incision was performed, as no intervention in the temporal area was desired/needed (Figure 1). We did not perform a classic coronal incision in any of the presented cases. This was due to the limited application of such an approach in FFS. It is performed only in cases where bone remodeling is required without any intervention in the hairline. Common surgeries performed in revision cases and primary cases were: hairline advancement, orbital shaving, browlift, and frontal bossing reduction. We performed endoscopic-assisted Type 3 surgery (reconstruction) with orbital shaving in seven patients (Figure 2 and Figure 3) and simple Type 1 osteoplasty (shaving) in three patients (Figure 4). One patient required revision surgery after Type 3 reduction.

The complication rate was low and temporary. One patient developed a biomaterial-associated infection of the scalp stabilization screw, which was removed on the 10th day after the surgery. Other patients developed unilateral temporal hematoma, which was drained on the 2nd day post-op (revision case). Shock-induced alopecia was diagnosed in another revision case, which was fully treated within 3 months after 6 rounds of PRF/Peptides injections. Full regrowth of the hair was observed at the time of the follow-up. No incidents of nerve paresthesia, injury to the frontal branch of the facial nerve, mucocele, facial paresis, cerebrospinal fluid leak, palpable contour deformities, or nonunion were reported.

## 4. Discussion

Facial feminization surgery is a distinctive part of surgical practice as it combines multidisciplinary knowledge and backgrounds rooted in cranio-maxillo-facial, plastic, and reconstructive surgery. Its evolution and form known today were a natural process since Paul Tessier set his miles stones in craniofacial surgery. Ousterchout’s research about male/female skull anthropometric differences performed in the 1980s was the first to target the transgender community directly, giving them hope for reaching their identity. Along with the technological development and incorporation of 3D virtual surgical planning procedures, they can be performed in a safer, time-consuming, and elegant manner [8,9] Patients usually do not need a hospital stay longer than 1–2 days. However, not every part of FFS may be calculated and automated. Indeed, reshaping of the skull features requires excellent training in cranio-maxillo-facial surgery. As a safe approach, the use of intraoral and subperiosteal dissections provides safe and reliable osteoplasty of every part of this complex region. “The projection of the soft tissues, understanding the subtle differences between cis-male and cis-female features, position of the brows, shape of the eyelid, canthal position, hairline design, and subdermal fat distribution are equally relevant in the final post-operational results. Therefore, it must be clearly stated that FFS surgery requires an in-depth understanding of clinical anatomy, anthropometry, skills in bone and plastic surgery, as well as artistic and esthetic touch. Transgender patients should be operated on at FFS-targeted centers that provide comprehensive care and a holistic approach [3,4] (Deschamps-Braly, 2019b; Ousterhout & Deschamps-Braly, 2019).

The group of patients presented here confirmed observations made in other research regarding feminization procedures of the upper third of the face. In the retrospective study performed by Rochlin et al. (2022) 25.5% of the cases were revision surgeries consisting of additional procedures due to previous under-correction [10]. In the analyzed material presented, 55% of the cases were revisions requiring additional procedures. However, our analysis was only restricted to the upper third.

When discussing bone remodeling of the upper face, one must take into consideration the proper approach to the skeleton, considering the hairline. In general, there are few approaches used in FFS of the upper third: standard bi-coronal, limited coronal incisions, coronal-pretrichial, and inverted-U trichial incisions [11]. When bone remodeling does not require hairline lowering or brow lifting, the classic coronal approach or limited coronal approach are then performed. This, however, is uncommon, as the vast majority of surgeries performed in FFS require re-shaping of the hairline and lowering and addressing the brows position in order to expose the widened orbits. Thus, the most common approach is pre-trichal hyperbeveled coronal incision, and such was usually done in our patients. Pansritum described an inverted U-trichial incision for simple supraorbital ridge shaving with or without augmentation [11]. This is a shortened version of the classical coronal approach and permits simple orbital ridge shaving without reconstruction. As most commonly performed forehead feminization surgeries require reconstruction, in minimal-access cases, we prefer our version of “short” incision, which is also U-shaped but not inverted. This approach provides much better access to the fronto-orbital region, enables reconstruction and not only simple shaving, and provides hairline lowering and/or trichophytic browlift (Figure 1c).

Regardless of the scalp incision design, one must keep in mind to avoid incisions in non-hair-bearing areas of the temples, as it is impossible to camouflage thereafter [10].

Endoscopic-assisted Type 3 forehead reduction with orbital shaving and hairline advancement in different extents with simultaneous temporal browlift with or without deep-plane midface lift was the most commonly performed surgery in our patients. This approach was the method of choice in most first-and-revision cases, as it provided feminization and rejuvenation. The use of trans nasal endoscopic light in order to set the borders of an anterior wall osteotomy minimizes the risk of injury to the dura and post-operational CFS leak reported in different studies (Figure 2) [12].

Scalp advancement requires sequentially performed galeotomies in order to mobilize and push the scalp forward. The general rule of galea scoring involves subgaleal-periosteal dissection up to the nuchal ridge, taking care to avoid injury to the occipital arteries. Galeal scoring is performed in a horizontal manner, and each galeotomy gives 1–2 mm of mobilization of the scalp [13]. The number of galeal scores depends on the extent of the lowering necessary; however, they should be planned judiciously without causing any harm to hair follicles. Usually, galeotomies are performed from the back of the scalp to the front (Video S1).

Video S1. Intraoperative view of the galeotomies. Distance between each should be 1–1.5 cm. Incisions should be made in a horizontal manner with care not to violate the skin and hair follicles. Each galeotomy provides 1–2 mm of scalp advancement.

**Figure 5:**
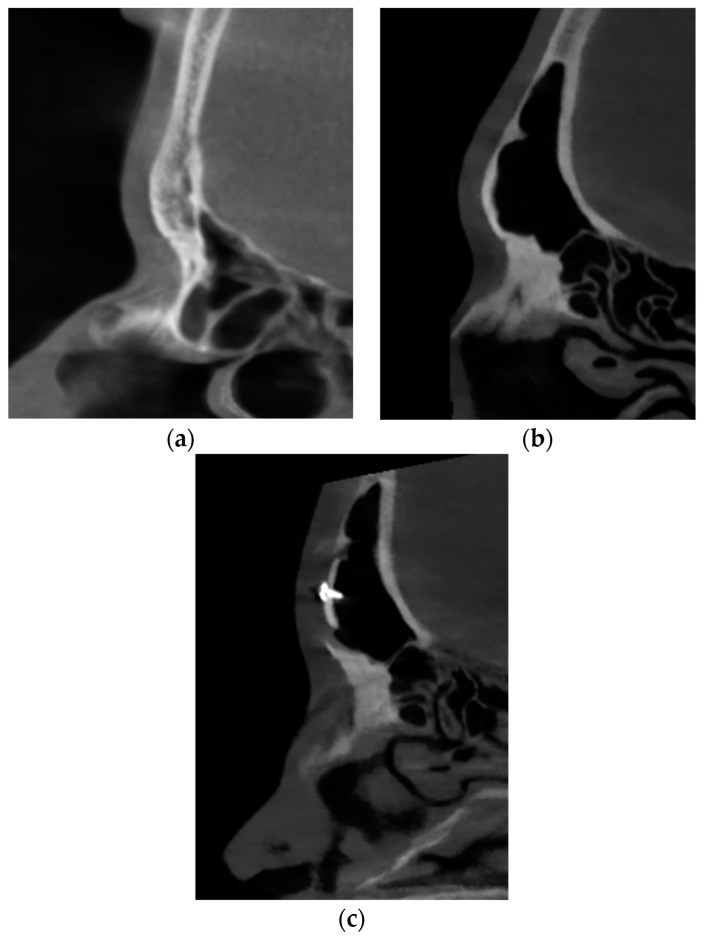
Sagittal sections of the frontal sinuses of two different patients who attended for the feminization of the upper third (not revision cases). (**a**) agenesis of the frontal sinus, which allowed for Type 1 forehead reduction. (**b**) large pneumatic frontal sinus, which required resection, remodeling, 5 mm setback, and osteosynthesis stabilization (Type 3). (**c**) the same sinus after feminization.

Forehead reduction in transgender facial surgery is commonly discussed in various publications. We do, however, prefer the classic approach described by Ousterhout. Type 3 surgery (reconstruction) was necessary in 75% of the cases. Surprisingly, revision cases were previously wrongly qualified patients for Type 1 reduction (bone burr). This was probably due to common undercorrection of the forehead, where Type 3 was really needed, but an unexperienced surgeon chose to burr down the cortical bone in a way not to open the sinus, leaving a thin wall. This was a frequently reported mistake, as Type 1 is only justified by an underdeveloped or lack of frontal sinus (Figure 4) [1]. In our cohort, only 10% of cases were Type 1 reduction, which generally agrees with the statistics published in other studies (Figure 5) [1,7,14,15].

We prefer conservative fixation of the anterior table with miniplates and miniscrews (typically micro-systems 1.2–1.5 mm for upper third reconstruction) as it provides sufficient stabilization and reduces the risk of nonunion. We do not use wires to stabilize the bone fragments, as was originally advised by Ousterhout, due to the undisputable superiority of stable osteosynthesis with regard to bone fragment stabilization. Similar observations were presented in the work of Lee et al. (2022). However, we opt for minimal use of biomaterials needed for stable fixation in order not to increase the risk of biomaterial-associated infections (preferably 2 miniplastes). We also do not find using titanium meshes in Type 3 surgery justified in other cases, which are more complicated, which is in contrast to the approach proposed by Bonapace–Potvin et al. (2022) [16]. In the work of Telang (2020), meshes were described as a substitute material for the reconstruction of the hypertrophic sinuses, where the anterior was resected and the defect closed with mesh only [15]. We do not support this technique, as leaving the sinus open without any bony anterior wall is completely unjustified, especially in aesthetic-reconstructive surgery. Meshes are also avoided in the protocol described by Maggio (2019) [14]. The author, however, prefers using wire fixation, as was originally postulated by Ousterhout. Maggio (2019) uses miniplates only when stabilization is not achieved by wires, showing that the topic of bone fragment stabilization in the non-mobile forehead is rather based on each author’s preferences and experience. If patients wish not to use titanium biomaterials, resorbable osteosynthesis is an option In selected cases.

We aim to preserve the frontal sinus mucosa by leaving the open nasofrontal duct. We ablate the sinus only in cases with a small sinus and an increased risk of mucocele and infections. In the presented report, no complications regarding the frontal sinus were reported.

Orbital shaving is as important as frontal bone setback. Male orbital volume is generally larger; however, women have a larger orbital opening compared to the rest of the face [17]. Undercorrection has been reported in the cohort of revision cases presented here and must have been addressed in secondary surgeries. Thus, much attention should be paid to the periorbital area when forehead osteoplasty procedures are planned. We aim at resecting the upper and upper-lateral orbital rim apertures. Similar to Maggio (2019), we are strongly convinced that burring down the upper aspect of the orbital rim may not provide sufficient feminization [14]. Especially as a properly dissected fronto-temporal area provides safe access to the upper and lateral aspects of the orbital aperture, wider resection is possible, which gives more feminine and balanced orbital widening than resection of the upper part only. More aggressive shaving of the upper-lateral part of the orbital aperture greatly enhances the effect of the temporal browlift during FFS. In our cohort, 7 patients who attended revision surgery complained about under resection of the orbital aperture, which was successfully corrected during revisions.

We advocate using suction drains. Some authors also do not use them, which, in our opinion, should be discussed [16]. We put at least 2 transverse suction drains: one under the frontal flap (in front of the incision) and a second caudally to the incision for 24–48 h. This prevents subgaleal hematoma and thus significantly reduces the incidence of traumatic alopecia and scalp necrosis [18].

**Figure 6:**
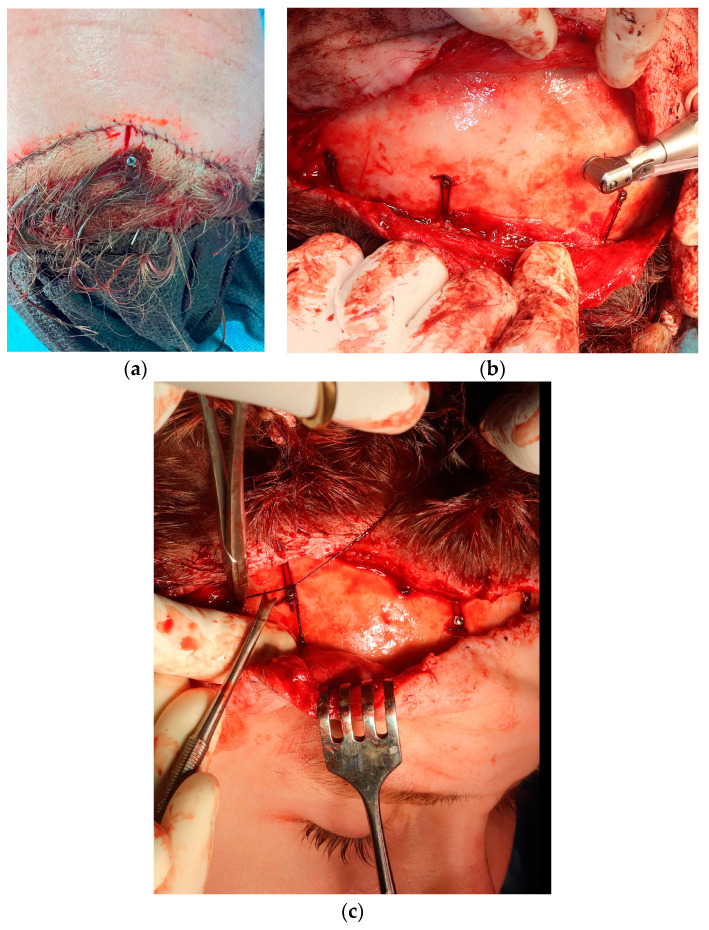
Intraoperative view of the two different techniques for scalp stabilization. (**a**) transcutaneous titanium screws placed temporarily directly on calvarial bone, 1.5–2 cm caudally to the incision line. Usually 4–5 screws of 2.0 × 15 mm are used. These can be easily identified and then removed 10–14 days later under local anesthesia. (**b**) permanent miniscrews placed monocortically under the skin-galea flap. Such screws may be used as both anchor points for scalp fixation sutures and browlift suspension sutures (**c**).

Scalp stabilization after its reposition usually requires some tension, and screw-based stabilization is always required. Closure should be firm; however, tension should be restricted to galea. The skin should be tensionless, while the galea should be stabilized in the calvarium. Otherwise, wound rupture, scarring, and hair loss may occur [13]. Generally, there are two commonly used techniques of scalp stabilization. The first is performed with the use of cranially fixated titanium screws piercing the skin-galeal flap 1.5–2 cm caudally to the incision line. These screws are kept for a period of 2–3 weeks, and then, after the scalp settlement, the screws are removed by a screwdriver (Figure 6). The second technique uses miniscrews placed on the calvarium. Sutures are placed subcutaneously, and tension is placed only on the galea. The first technique may be used in advancements with low to moderate tension, as stabilization and screw removal are straightforward. However, if scalp advancement is significant and increased tension is to be expected, calvarium-based miniscrews are advised (Figure 5). This reduces the risk of necrosis, alopecia, infection, and bad scars. Alternative fixations are endotines or cortical tunnels [17].

Another observation made in this analysis was that browlift procedures were not specifically addressed in the cohort who attended revision surgery. It was surprising, as browlifting is an inherent part of facial feminization. The position of the brows and their lateral tails is one of the most important factors distinguishing the male and female peri-orbital areas, even without bone reduction surgery. Osteoplasty of the upper and upper-lateral orbital rim is performed in order to widen the orbital aperture and expose the eyeballs, but without any form of browlift, this effect cannot be achieved as soft tissues will remain in their place or will even droop due to bone deficiency after reduction. Surprisingly, 9 out of 11 revision cases required a browlift during their second surgery. We usually perform temporal browlifts during scalp transection and hairline advancement with deep-plane cheek lifts, canthoplasty, and eyelid surgery. We performed both hard and soft tissue feminization during one-stage surgery, similarly to the protocol described by Maggio (2019) [14]. However, we do not advise a hair transplant at the time of the main surgery, as injury to the scalp and frontal flap may affect graft healing and stability. This is in contrast to the study published by Capitain et al. (2017), as they encourage performing FUE grafting at the time of the facial surgery [6]. The authors justify this technique by introducing the posterior-coronal flap, which in theory provides hairline lowering and the simultaneous use of strip hair follicles. This seems illogical, as resection of the skin behind the hairline can produce nothing but forehead lengthening, which is then masked by grafts. Careful planning of the hairline incision with regards to the full-feminization plan is crucial. It is completely unjustified to perform a coronal incision placed behind a hairline if the patient requires hairline-lowering surgery. Especially in revision cases, the risk of alopecia between two incisions is increased. Such observations were described in the report of Dechamps–Braly (2019) [10] (2 cases), and so they were diagnosed in one of our patients, where revision of the Type 3 surgery was necessary due to insufficient setback, deformity, and a lack of addressing the hairline during the first surgery. Shock hair loss may develop in 10% of scalp advancement cases, but it increases significantly in secondary procedures. Especially when a second, different incision design is necessary for hairline lowering, which results in the formation of two scars that impede blood supply to the hair follicles. Therefore, it is an absolute must to address all the issues judicially during the first surgery and not increase the risk of unwanted complications. Shock hair loss is usually temporary, and hair regrowth is expected after a few weeks or months. Platelet-rich fibrin (PRF) and hair-stimulating peptide injections every two weeks provide sufficient boosters for regrowth, as was fortunately observed in our patient.

We do advise hair transplants 6–12 months after scalp advancement for a more predictable outcome and better addressing the temple hollowing and potential scar, similarly to the protocols described by Dechamps–Braly (2019) [3,4]. Last but not least, the gold standard of hair grafting is the follicular unit extraction (FUE) technique. As such, it requires the patient’s prone position for follicular harvesting from the occipital area. Not only would it require changing the patient’s position during feminization surgery (supine position), but it would also result in the unjustified elongation of general anesthesia. Taking into account all the aforementioned arguments, delayed hair transplant as an adjunctive treatment that can be comfortably performed under local anesthesia is the most advantageous, effective, and safest.

Soft and hard tissue relationships are of great importance in any facial surgery. However, in FFS, one must aim at the feminine aspect by reducing osteoplasties and, hence, considering the physiology and anatomy of the aging face. Issues related to anti-aging procedures go beyond the scope of this manuscript. What is more, upper-third feminization surgery is usually also a solution for aging processes in the upper face through browlifts. Nevertheless, a few remarks should be made regarding lifting procedures as such. Contrary to the protocols described by Maggio, we advise addressing the mid- and lower face and neck soft tissues 6–12 months after bone remodeling and not simultaneously [14]. Especially in the aging face. In our experience, face and neck lifts, liposculpting, and other surgeries are far more powerful after the final settlement of these tissues on the remodeled bone framework, and by that, the risk of under correction (typically in facelifts) is far less plausible. When considering facial feminization surgery, upper-third feminization may greatly deal with aging in this area during one-stage surgery. However, in the case of the lower third, we strongly recommend a 2-stage approach for better re-draping of the soft tissues after bone reduction procedures.

## 5. Conclusions

The authors’ preferred technique, ‘whole-in-one’ upper face feminization by modified bi-coronal incision with frontal trichophytic hyper-beveled incision, provides sufficient insight into the frontal bone and orbital region, the desired forehead osteoplasty, and the most efficient insight into the temporal area, enabling safe dissection between fasciae, ligamentous adhesion removal, and periosteal attachment, providing full soft and hard tissue feminization during one surgery. The approach is suitable for revision surgery as it is for the first-time upper-face full feminization. The study’s findings highlight the importance of additional training in facial surgery for transgender patients in order to offer them comprehensive care within a single surgical facility. This will have a significant impact on patient safety, aesthetic outcomes, and overall satisfaction.

## Figures and Tables

**Table 1:** Patients included into the study and procedures performed before elsewhere and reasons for eventual secondary surgery.

Patient	Age	Type 2	Type 2	Type 3	Type 4	Orbital Shaving	Hairline Advancementt	Browlift	Complaint	Commentary
1	42	yes	no	no	no	no	no	no	yes	insufficient forehead and orbital reduction, hairline
2	23	no	no	no	no	no	no	no	no	no
3	19	no	no	no	no	no	no	no	no	no
4	17	no	no	no	no	no	no	no	no	no
5	20	no	no	no	no	no	no	no	no	no
6	18	no	no	no	no	no	no	no	no	no
7	24	no	no	no	no	no	no	no	no	no
8	31	yes	no	no	no	no	no	no	yes	insufficient forehead and orbital reduction, hairline
9	45	no	no	no	no	no	yes	yes	yes	lack of bone reduction at all
10	23	no	no	no	no	no	no	no	no	no
11	23	no	no	no	no	no	no	no	no	no
12	45	yes	no	no	no	yes	no	no	yes	insufficient forehead and orbital reduction, hairline
13	51	yes	no	no	no	no	no	no	no	hairline
14	34	no	no	no	no	no	no	yes	yes	insufficient forehead and orbital reduction, hairline
15	32	no	no	no	no	no	yes	no	yes	insufficient forehead and orbital reduction, hairline
16	45	no	no	no	no	no	yes	yes	yes	insufficient forehead and orbital reduction, hairline
17	21	no	no	no	no	no	no	no	no	no
18	19	no	no	no	no	no	no	no	no	no
19	35	no	no	no	no	no	no	no	no	no
20	19	no	no	yes	no	no	no	yes	yes	insufficient forehead and orbital reduction, hairline, browlift

**Table 2:** Patients included into the study and procedures performed at our clinic.

Patient	Type 1	Type 2	Type 3	Type 4	Orbital Shaving	Hairline Advancement	Browlift	Complaint	Complication	Type of Complication
1	no	no	no	no	no	no	no	no	yes	Screw infection
2	yes	no	no	no	yes	yes	yes	no	no	x
3	no	no	yes	no	yes	yes	yes	no	no	x
4	no	no	no	no	yes	yes	yes	no	no	x
5	no	no	yes	no	yes	yes	yes	no	yes	Shock hair loss
6	no	no	yes	no	yes	yes	yes	no	no	x
7	no	no	yes	no	yes	yes	yes	no	no	x
8	no	no	yes	no	yes	yes	yes	no	no	x
9	no	no	yes	no	yes	yes	yes	no	no	x
10	no	no	yes	no	yes	yes	yes	no	no	x
11	yes	no	no	no	yes	yes	yes	no	no	x
12	no	no	yes	no	yes	yes	yes	no	yes	Temporal hematoma
13	no	no	no	no	yes	yes	no	no	no	x
14	no	no	yes	no	yes	yes	yes	no	no	x
15	no	no	yes	no	yes	yes	yes	no	no	x
16	no	no	yes	yes	yes	yes	yes	no	no	x
17	no	no	yes	no	yes	yes	yes	no	no	x
18	no	no	yes	no	yes	yes	yes	no	no	x
19	yes	no	no	no	yes	no	yes	no	no	x
20	no	no	yes	no	yes	yes	yes	no	no	x

**Table 3:** Detailed upper third FFS procedures decision tree.

**Hairline**	High	Scalp advancement surgery
	Low	Consider coronal/temporal browlift only
**Receding hairline**	Yes	Correction during scalp advancement
	No	In case of scalp advancement consider less invasive incision
**Widow’s peak**	Present	Remove for better scar camouflage
	Not Present	x
**Medial brow ptosis**	Yes	Addressed during scalp advancement and/or coronal browlift
	No	x
**Lateral brow ptosis**	Yes	Temporal lift during scalp advancement or other closed techniques
	No	x
**Negative vector of the eye**	Yes	Addressed during temporal lift. Consider canthopexy/canthoplasty along with midface lift and feminizing upper/lower blepharoplasty
	No	Simple upper/lower blepharoplasty if needed
**Orbital shaving required**	Yes	Osteoplasty with piezosurgery/pineapple bone bur
	No	x
**Frontal bossing**	Present	Ousterhout Type 1, 2, 3 or 4 forehead procedure
	Not present	x

## Data Availability

Data are contained within the article.

## Supplementary Materials

The following supporting information can be downloaded at: https://www.mdpi.com/article/10.3390/medicina60010120/s1, Video S1.
